# Generalized L-Shaped Nested Array Concept Based on the Fourth-Order Difference Co-Array

**DOI:** 10.3390/s18082482

**Published:** 2018-08-01

**Authors:** Lei Zhang, Shiwei Ren, Xiangnan Li, Guishan Ren, Xiaohua Wang

**Affiliations:** 1 School of Information and Electronics, Beijing Institute of Technology, 5 South Zhongguancun Street, Haidian District, Beijing 100081, China; zhl666@bit.edu.cn (L.Z.); renshiwei@bit.edu.cn (S.R.); 3120170367@bit.edu.cn (X.L.); 2 Oil Production Technology Institute of Da Gang Oilfield Company, No. 1278, Xingfu Road, Haibin Street, Binhai New District, Tianjin 300280, China; rengshan@petrochina.com.cn

**Keywords:** fourth-order difference, two-dimensional sparse array, quasi-stationary signal

## Abstract

In this paper, a generalized L-shaped nested array based on the fourth-order difference co-array is proposed for two-dimensional (2D) directions’ estimation. The new structure framework makes full use of the physical sensor locations to form a virtual uniform rectangular array (URA) as large as possible. As it utilizes the fourth-order difference instead of the traditional second-order difference result, this structure framework can acquire a much higher degree-of-freedom (DOF) than the existing 2D sparse arrays. The proposed structures have two advantages. One is that the subarrays can be chosen as any nested-class arrays, which makes the sparse array design more flexible. We can choose arbitrary subarray structures for DOF enhancement purposes. Another advantage is that the relative position of two subarrays can be set as any integral multiple of half wavelength. This means that two subarrays can be located as far as possible so that the relative influence between two physical subarrays can be ignored. The DOFs of several typical generalized L-shaped nested arrays (GLNAs) are compared in this paper. By setting the subarrays as different types and the relative position as a special value, a special GLNA is presented. Simulations show that GLNAs have obvious superiority in 2D direction-of-arrival estimation.

## 1. Introduction

In the past decades, two-dimensional (2D) planar arrays have been widely used in the field of beamforming, imaging, communication and radar [1]. The traditional configurations people usually use are the uniform rectangular arrays (URAs) and hexagonal arrays. Such kinds of arrays have physical sensors uniformly located on the rectangular grids, so that the 2D subspace estimation algorithms, such as the 2D ESPRIT method [2], can be directly applied on the received data. Although URA can obtain robust direction-of-arrival (DOA) estimation, the number of detectable sources is limited in the order of O(N). The mutual coupling effect between the adjacent sensors influences the performance of URAs seriously, as well. Therefore, the sparse arrays with larger spacings between the adjacent sensor pairs have attracted much attention in the last five years.

The nested array [3] and coprime array [4,5] are important one-dimensional (1D) sparse arrays based on the concept of the second-order difference co-array (SODC), which can detect $O(N^{2})$ sources with only *N* sensors. To further reduce the mutual coupling arising from the dense uniform linear array (ULA) in the nested array, a super nested array [6,7] is proposed to remove the dense physical sensors from the sparse ULA area. For the purpose of degree-of-freedom (DOF) enhancement and mutual coupling reduction, augmented nested arrays [8] are proposed, as well. Based on the coprime array, the DOF of which is much lower than that of the nested array, several optimized structures are proposed and verified to be effective in increasing DOF, such as the coprime array with compressed inter-element spacing (CACIS) [9], the coprime array with displaced subarrays (CADiS) and the coprime array with multi-period subarrays (CAMpS) [10].

Inspired by the 1D sparse array design, a few planar sparse arrays (PSAs) have been proposed recently for 2D sub-sampling and signal recovery. Some well-known PSA geometries include the L-shaped nested array [11,12], 2D coprime array, 2D nested array [13,14], billboard arrays, open box arrays [15] and hourglass arrays [16]. Among all these structures, the L-shaped nested array and 2D co-prime array are provided mainly to resolve the ambiguity problem and implement fast DOA estimation. The other PSA structures make use of the SODC concept to acquire the hole-free virtual planar array with DOF increasing from O(M+N) to O(MN). Although the performance is improved significantly, there is still much space for DOF enhancement.

Recently, the fourth-order difference co-array (FODC) concept was proposed and utilized for 1D DOA estimation [17], which is verified to be effective in DOF enhancement. The new sparse arrays based on the 2qth-order cumulant [18] and especially the fourth-order cumulant [19] are the 2q-level nested array and sparse array extension with the fourth-order difference co-array enhancement based on the two-level nested array (SAFOE-NA). Another algorithm that can generate FODC makes use of the quasi-stationary (QS) signal properties to acquire the virtual FODC array. As many speech and audio signals have been proven to be quasi-stationary, such an FODC construction scheme has great practical significance. It is verified that by exploiting the FODC methods, the DOF of the 1D sparse arrays can be increased significantly. Nowadays, the four-order difference concept is only used in 1D sparse array design. For the 2D sparse array, there has been no study conducted so far.

In this paper, the generalized L-shaped nested arrays (GLNAs) based on the FODC concept are proposed for DOF enhancement. The new 2D configuration is a generalized framework including two nested-class subarrays positioned at an arbitrary distance. The nested-class arrays guarantee that a cross-shaped structure can be formed in SODC. Therefore, a virtual URA with the center at the origin is constructed in the FODC. As the FODC concept instead of SODC is utilized in the new GLNA construction, the number of detectable sources will be increased greatly. Since the subarrays can be chosen as any kind of existing nested arrays, more choices are provided in sparse array design. By setting the subarrays with a large 1D virtual consecutive range and sparse array structure, GLNA can achieve a high number of DOFs. Another advantage of GLNA is that the relative 2D position of the subarrays can be chosen as any integer times the minimum spacial interval *d*. Since relative distance can be set as large as possible, the influence between two physical subarrays can be greatly reduced. To make complete comparisons among all GLNAs, we provide the expressions of URA size and list the values of several different GLNAs. Furthermore, a special GLNA with the highest number of DOFs is presented and analyzed.

This paper is organized as follows. The QS signal model and FODC concept are presented in Section 2. The proposed generalized L-shaped nested array is proposed in Section 3. The DOF characteristics are summarized. Several GLNAs are compared to other SODC-based PSAs in the aspect of DOF. In Section 4, a special GLNA (sGLNA) is developed and analyzed, showing its great performance improvement in DOF enhancement. Simulation results are shown in Section 5, and the conclusions are drawn in Section 6. Notations: We denote vectors and matrices by using lower-case and upper-case bold characters, respectively. Specifically, ${\left(.\right)}^{*}$ denotes complex conjugate, whereas ${\left(.\right)}^{T}$ and ${\left(.\right)}^{H}$ respectively denote the transpose and conjugate transpose of a matrix or vector. vec(.) implies the vectorization operator, which turns a matrix into a column vector. E(.) is the statistical expectation operator. ${\left|.\right|}_{\ell_{2}}$ is the Frobenius norm of the 2D vector in it.

## 2. Signal Model

Consider *D* uncorrelated narrowband QSsignals $s_{i}\left(t\right),i=1,\cdots ,D$ from direction $\left(\theta_{i},\phi_{i}\right)$ impinging on an *N*-sensor planar array with physical sensors located at $S=[n_{1};n_{2};\cdots ;n_{N}]d$. $n_{j}=\left(n_{jx},n_{jy}\right)$ is the *j*-th normalized sensor location for j=1,2,⋯,N. *d* is the unit spacing between two sensors, which is also the length unit of the x and y Cartesian axes, where the arrays are placed. The value of *d* is set to be half the minimal wavelength λ. For convenience, *d* is omitted in the following discussions. Assume that the source signals $s_{i}\left(t\right),i=1,\cdots ,D$ are wide-sense quasi-stationary within the frame length $\tilde{P}$. Denote $s_{i}\left(p,k\right)$ as the *i*-th source signal at the *k*-th snapshot within the *p*-th frame. Then, the power $\sigma_{p,i}^{2}=E\left\{s_{i}\left(p,k\right)s_{i}^{*}\left(p,k\right)\right\}$ for $k\in \{p\cdot \tilde{P},p\cdot \tilde{P}+1,\cdots ,\left(p+1\right)\cdot \tilde{P}-1\}$ can be approximated by [20]:

(1) $$\begin{array}{c} \sigma_{p,i}^{2}\approx \frac{1}{\tilde{P}}\sum_{k=p\cdot \tilde{P}}^{\left(p+1\right)\cdot \tilde{P}-1}s_{i}\left(p,k\right)s_{i}^{*}\left(p,k\right) \end{array}$$

Then, the received signals from *N* sensors at the *k*-th snapshot within the *p*-th frame can be represented as:

(2) $$\begin{array}{c} x_{S}\left(p,k\right)=\sum_{i=1}^{D}a_{S}\left(\theta_{i},\phi_{i}\right)s_{i}\left(p,k\right)+w_{S}\left(p,k\right), \end{array}$$

where the element of $a_{S}\left(\theta_{i},\phi_{i}\right)$ for the *i*-th source impinging on the grid $\left(n_{jx},n_{jy}\right)$ is $e^{j2\pi d/\lambda (sin\theta_{i}cos\phi_{i}n_{jx}+sin\theta_{i}sin\phi_{i}n_{jy})}$; $w_{S}\left(p,k\right)$ is the wide-sense stationary noise vector.

By taking all the *K* snapshots in the *p*-th frame into consideration, the correlation matrix is given by:

(3) $$\begin{array}{c} R_{xx}\left(p\right)=E\left\{x_{S}\left(p,k\right)x_{S}{\left(p,k\right)}^{H}\right\}=AR_{ss}\left(p\right)A^{H}+\sigma_{n}^{2}I, \end{array}$$

where $A=[a_{S}\left({\bar{\theta }}_{1},{\bar{\phi }}_{1}\right),\cdots ,a_{S}\left({\bar{\theta }}_{D},{\bar{\phi }}_{D}\right)]$ is the array manifold; $R_{ss}\left(p\right)=diag\left\{\sigma_{p,1}^{2},\sigma_{p,2}^{2},\cdots ,\sigma_{p,D}^{2}\right\}$ is the source covariance matrix; $\sigma_{n}^{2}$ is the noise power; $I\in C^{N\times N}$ is the identity matrix. Vectorizing $R_{xx}\left(p\right)$ yields:

(4) $$\begin{array}{c} z\left(p\right)=\mathrm{vec}\left\{R_{\mathrm{xx}}\left(p\right)\right\}=\left(A^{*}⊗A\right)\tilde{s}\left(p\right)+l_{N}\left(p\right), \end{array}$$

where $\tilde{s}\left(p\right)={\left[\sigma_{p,1}^{2},\sigma_{p,2}^{2},\cdots ,\sigma_{p,D}^{2}\right]}^{T}$ can be viewed as the virtual source vector; $l_{N}\left(p\right)=vec\left\{\sigma^{2}\left(p\right)I\right\}\in C^{N^{2}\times 1}$ is the noise vector. Assume two sensor location matrices as $S_{1}=\left[n_{11};n_{12};\cdots ;n_{1N_{1}}\right]$ and $S_{2}=\left[n_{21};n_{22};\cdots ;n_{2N_{2}}\right]$, the numbers of sensors if which are $N_{1}$ and $N_{2}$, respectively. Define the sensor location difference operation as $Diff\left(S_{1}-S_{2}\right)=\left[\left(n_{11}-n_{21}\right);\left(n_{11}-n_{22}\right);\cdots ;\left(n_{11}-n_{2N_{2}}\right);\left(n_{21}-n_{21}\right);\left(n_{21}-n_{22}\right);\cdots ;\left(n_{21}-n_{2N_{2}}\right);\cdots ;\left(n_{1N_{1}}-n_{21}\right);\left(n_{1N_{1}}-n_{22}\right);\cdots ;\left(n_{1N_{1}}-n_{2N_{2}}\right)\right]$. Then, the Kronecker product $\left(A^{*}⊗A\right)$ is an equivalent manifold matrix, whose virtual sensors are located at $\bar{C}=Diff\left(S-S\right)$. It is actually an SODC with the virtual elements at the difference positions of the physical sensor locations.

As the assumption given by [20], $\sigma_{p,i}^{2},i=1,\cdots ,D$ are wide-sense stationary and uncorrelated with each other. Denote the self-correlation of $\sigma_{p,i}^{2}$ as ${\tilde{\sigma }}_{i}^{2}=E\left\{{\left(\sigma_{p,i}^{2}-E\left\{\sigma_{p,i}^{2}\right\}\right)}^{2}\right\}$. Then, SODC can be conducted again to form FODC after the zero-mean equivalent impinging sources have been generated.

Denote the expectation over *P* frame as E[z(p)]. We have:

(5) $$\begin{array}{c} E\left[z\left(p\right)\right]=\left(A^{*}⊗A\right)E\left[\tilde{s}\left(p\right)\right]+l_{N}\left(p\right), \end{array}$$

Subtracting the above result from z(p), we can obtain:

(6) $$\begin{array}{c} \bar{z}\left(p\right)=z\left(p\right)-E\left[z\left(p\right)\right]\approx \tilde{A}\bar{s}\left(p\right), \end{array}$$

where $\tilde{A}=\left(A^{*}⊗A\right)$ with the *i*-th column as $a({\bar{\theta }}_{i},{\bar{\phi }}_{i})$ and $\bar{s}\left(p\right)=\tilde{s}\left(p\right)-E\left[\tilde{s}\left(p\right)\right]$.

As $\bar{s}\left(p\right)$ can be viewed as a zero-mean virtual signal vector, the difference co-array concept can be applied again. Similarly to (3), the correlation matrix of the virtual received signals $\bar{z}\left(p\right)$ over *P* frames can be represented as:

(7) $$\begin{array}{c} R_{zz}=E\left\{\bar{z}\left(p\right){\bar{z}}^{H}\left(p\right)\right\}=\tilde{A}R_{\bar{s}\bar{s}}{\tilde{A}}^{H}, \end{array}$$

where $R_{\bar{s}\bar{s}}=diag\left\{{\tilde{\sigma }}_{1}^{2},\cdots ,{\tilde{\sigma }}_{D}^{2}\right\}$.

Performing the vectorization again, one has:

(8) $$\begin{array}{c} y=\mathrm{vec}\left\{R_{\mathrm{zz}}\right\}=\left({\tilde{A}}^{*}⊗\tilde{A}\right)\hat{s}, \end{array}$$

where $\left({\tilde{A}}^{*}⊗\tilde{A}\right)$ is the virtual manifold matrix with the virtual sensors located at $\hat{C}=Diff(\bar{C}-\bar{C}$); $\hat{s}=vec\left(R_{\bar{s}\bar{s}}\right)={\left[{\tilde{\sigma }}_{1}^{2},\cdots ,{\tilde{\sigma }}_{D}^{2}\right]}^{T}$, is the equivalent source signal vector. Perform the 2D spatial smoothing algorithm [21] and the 2D subspace algorithms such as 2D unitary-ESPRIT [22] and 2D MUSIC [23] directly on y; 2D DOA estimations can be obtained.

## 3. The Proposed L-Shaped Nested Arrays

Different from the existing L-shaped nested array [11,12] and other second-order difference co-array-based structures [13,14,16], the new 2D sparse arrays proposed in this paper are based on the fourth-order difference co-array concept. That means as long as a consecutive URA can be extracted from FODC, the 2D spatial smoothing algorithm can be made use of directly on it. Therefore, in this paper, we focus on designing a kind of 2D sparse array that can generate a URA area as large as possible in FODC.

Since the 2D sparse arrays we will develop can be grouped into a unified framework, we first introduce the generalized concept and then compare the DOF of several sub-structures in the framework. For convenience, in the following sections, we use the normalized sensor locations to represent the actual physical positions, i.e., *d* is omitted in the following discussions.

### 3.1. Generalized L-Shaped Nested Array

Note that *d* is omitted in the following discussions for convenience. Then, the generalized L-shaped nested array can be described by the definition shown below.

**Definition** **1.** *(Generalized L-shaped nested array (GLNA)): Given two 1D nested-class arrays described by the matrix $C_{h}$ and $C_{v}$, where $C_{h}=\left[s_{h1};s_{h2};\cdots ;s_{hN_{x}}\right]$ and $C_{v}=\left[s_{v1};s_{v2};\cdots ;s_{vN_{y}}\right]$, the sensor numbers of these two arrays are $N_{x}$ and $N_{y}$, respectively. Assume $N=N_{x}+N_{y}$, then a generalized L-shaped nested array is an N-sensor 2D planar sparse array located on the grids, which is denoted as follows:*

(9) $$S_{\mathrm{GLNA}}=\left[H;V\right]$$

*where $H=[\left(s_{h1},0\right);\left(s_{h2},0\right);\cdots ;\left(s_{hN_{x}},0\right)]$ and $V=[\left(L_{x},L_{y}+s_{v1}\right);\left(L_{x},L_{y}+s_{v2}\right);\cdots ;\left(L_{x},L_{y}+s_{vN_{y}}\right)]$. $L_{x}$ and $L_{y}$ can be any integers.*

**Figure 1:**
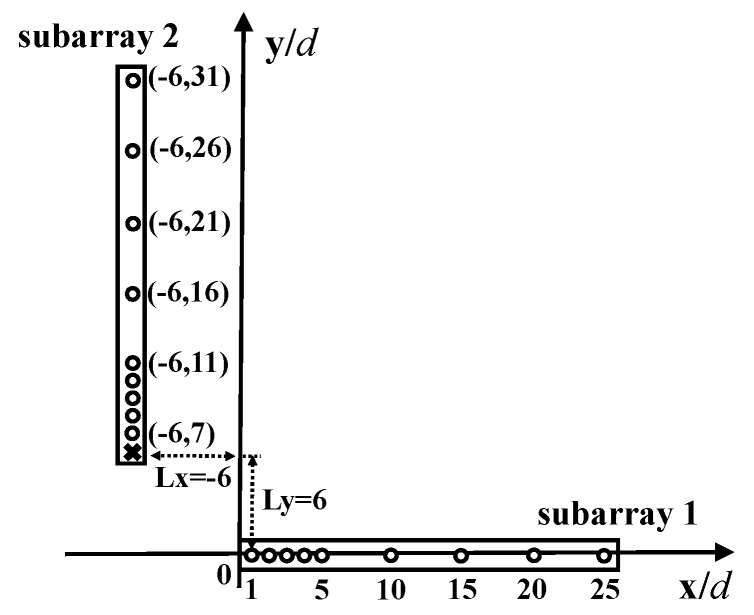
A generalized L-shaped nested array (GLNA) with two classic nested arrays (CNAs) (GLNA-CNA) having the same parameters $\left(N_{d},N_{s}\right)=\left(4,5\right)$ distributed in the x, y directions with (0,0) and (−6,6) as the reference. The circles represent physical sensors. The cross is a hole.

It can be summarized from the definition that a GLNA consists of two 1D nested-class arrays located in the horizontal and vertical direction, respectively. H and V are the sensor locations of two subarrays, which can be any kind of aforementioned nested array, such as the classic nested arrays (CNAs), super nested arrays (SNAs) and augmented nested arrays (ANAs). The relative position parameter pair in this definition, $\left(L_{x},L_{y}\right)$, of the two subarrays is arbitrary. To have a better idea of the GLNA configuration, we take an example by setting CNAs as subarrays. As shown in Figure 1, two 1D CNAs with $C_{h}=C_{v}=\left[1;2;3;4;5;10;15;20;25\right]$ are distributed on the x-axis, and line x=−6. The reference points of the two subarrays are (0,0) and (−6,6) with the relative displacement as $\left(L_{x},L_{y}\right)=\left(-6,6\right)$. Therefore, the vertical sensors are located on V=[(−6,7);(−6,8);(−6,9);(−6,10);(−6,11);(−6,16);(−6,21);(−6,26);(−6,31)]. The horizontal subarray lies on positions H=[(1,0);(2,0);(3,0);(4,0);(5,0);(10,0);(15,0);(20,0);(25,0)]. The total number of sensors in this GLNA is 18. Note that in the following structure, as length unit *d* is defined to be the half-wavelength, it is clear that the units of the x and y Cartesian axes of the array geometry are also in half wavelengths.

Denote the second-order and fourth-order self-difference of any sparse array S as ${\bar{C}}_{S}=Diff\left(S-S\right)$ and ${\hat{C}}_{S}=Diff\left({\bar{C}}_{S}-{\bar{C}}_{S}\right)$, respectively. The SODC and FODC of GLNA have the following conclusions.

**Property** **1.** *For two 1D subarrays $C_{h}$ and $C_{v}$ of GLNA defined by (9), their second-order self-differences are denoted as ${\bar{C}}_{C_{h}}$ and ${\bar{C}}_{C_{v}}$. If there exists consecutive lags in their self-difference arrays, i.e., $\left[-L_{h};\left(-L_{h}+1\right);\cdots ;\left(L_{h}-1\right);L_{h}\right]$ is a part of ${\bar{C}}_{C_{h}}$ and $\left[-L_{v};\left(-L_{v}+1\right);\cdots ;\left(L_{v}-1\right);L_{v}\right]$ is a part of ${\bar{C}}_{C_{v}}$, then the SODC of GLNA must include a cross-shaped structure distributed at $S_{d}=\left[S_{h};S_{v}\right]$ with $S_{h}=\left[\left(-L_{h},0\right);\left(-L_{h}+1,0\right);\cdots ;\left(L_{h}-1,0\right);\left(L_{h},0\right)\right]$ and $S_{v}=\left[\left(0,-L_{v}\right);\left(0,-L_{v}+1\right);\cdots ;\left(0,L_{v}-1\right);\left(0,L_{v}\right)\right]$. The FODC of GLNA contains a central URA with a minimum aperture of $\left(2L_{h}+1\right)\times \left(2L_{v}+1\right)$.*

**Proof.**  First, let us consider the SODC of GLNA. According to the expression of GLNA in (9), SODC can be written as:

(10) $${\bar{C}}_{S}=\left[D_{h};D_{v};D_{hv};D_{vh}\right],$$

with $D_{h}=Diff\left(H-H\right)$ and $D_{v}=Diff\left(V-V\right)$ as the self-differences of subarrays H and V. ${\left[D_{hv},D_{vh}\right]}^{T}$ is the cross-difference. As H is a nested-class array located on the x-axis, its self-difference is still a linear array with the form of $D_{h}=\left[{\bar{C}}_{C_{h}},\vec{0}\right]$. Since the assumption that there exist consecutive lags in the self-difference arrays of each subarray, we conclude that $S_{h}=[\left(-L_{h},0\right);\left(-L_{h}+1,0\right);\cdots ;$$\left(L_{h}-1,0\right);\left(L_{h},0\right)]$ is a part of matrix $D_{h}$. Similarly, the self-difference result of V is a linear structure at the y-axis located at $D_{v}=\left[\vec{0},{\bar{C}}_{C_{v}}\right]$. Its array geometry also has a consecutive segment included in the center, i.e., the matrix $S_{v}=\left[\left(0,-L_{v}\right);\left(0,-L_{v}+1\right);\cdots ;\left(0,L_{v}-1\right);\left(0,L_{v}\right)\right]$ is a part of $D_{v}$. Then, we turn to $D_{vh}$. From the definition of H and V, it is not difficult to obtain that $D_{vh}$ is a matrix, the elements of which can be denoted as $\left(-s_{h,i}+L_{x},s_{v,j}+L_{y}\right)$. Here, $s_{h,i}$ and $s_{v,j}$ are the *i*-th and the *j*-th elements of the matrix $C_{h}$ and $C_{v}$, respectively. Such a matrix is the position of the cross-difference array, which is in fact several copies of the vertical subarray with the *i*-th reference points positioned at $\left(-s_{h,i}+L_{x},L_{y}\right)$ where $s_{h,i}$ is the *i*-th element of the matrix $C_{h}$. Another cross-difference matrix $D_{hv}$ is the reverse of $D_{vh}$. Therefore, it generates the same number of vertical subarrays symmetrically. In conclusion, no matter what type of subarray in GLNA is, as long as each of them can generate a consecutive segment by making the self-difference, it is definite that SODC will contain a cross-shaped structure at the x-axis and y-axis, i.e., matrix $\left[S_{v},S_{h}\right]$ is a part of matrix ${\bar{C}}_{S}$.

Then, we extract the cross structure out and make the difference again to generate part of FODC. Let $S_{d}=\left[S_{v};S_{h}\right]$. Again, we have matrix $Diff\left(S_{d}-S_{d}\right)=\left[Diff\left(S_{v}-S_{v}\right);Diff\left(S_{h}-S_{h}\right);Diff\left(S_{v}-S_{h}\right);Diff\left(S_{h}-S_{v}\right)\right]$ is a part of ${\hat{C}}_{S}=Diff\left({\bar{C}}_{S}-{\bar{C}}_{S}\right)$. From the form of $S_{h}$ and $S_{v}$, we deduce that $Diff\left(S_{h}-S_{h}\right)=\left[\left(-2L_{h},0\right);\left(-2L_{h}+1,0\right);\cdots ;\left(2L_{h}-1,0\right);\left(2L_{h},0\right)\right]$, $Diff\left(S_{v}-S_{v}\right)=\left[\left(0,-2L_{v}\right);\left(0,-2L_{v}+1\right);\cdots ;\left(0,2L_{v}-1\right);\left(0,2L_{v}\right)\right]$ and $Diff\left(S_{v}-S_{h}\right)=Diff\left(S_{h}-S_{v}\right)=\left[\left(-L_{h},-L_{v}\right);\left(-L_{h},-L_{v}+1\right);\cdots ;\left(-L_{h},L_{v}\right);\left(-L_{h}+1,-L_{v}\right);\cdots ;\left(L_{h},L_{v}\right)\right]$. It contains a URA with the size of $\left(2L_{h}+1\right)\times \left(2L_{v}+1\right)$. ☐

**Figure 2:**
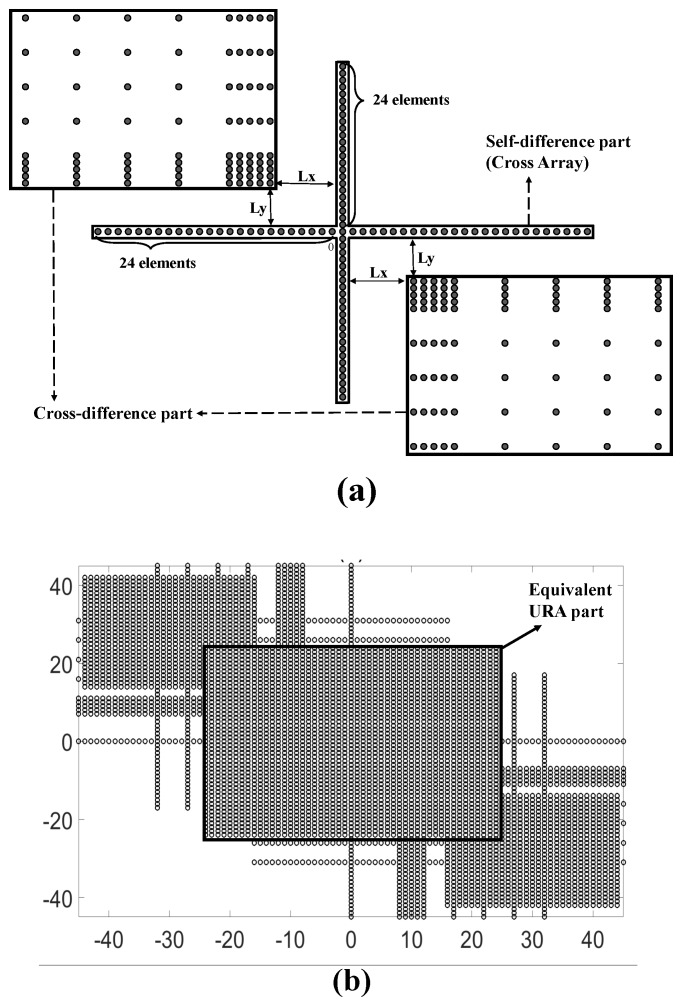
Difference co-arrays of the GLNA in Figure 1. (**a**) second-order difference co-array (SODC) (**b**) fourth-order difference co-array (FODC) (note that the unit of length in this figure is *d*). URA, uniform rectangular array.

Figure 2 illustrates the SODC and FODC of GLNA in Figure 1. As the two subarrays in GLNA are the same classic nested arrays with the self-difference being hole-free, i.e., ${\bar{C}}_{C_{h}}={\bar{C}}_{C_{v}}=[-L_{h};-L_{h}+1;$$\cdots ;L_{h}]$ with $L_{h}=24$, the SODC in Figure 2a possesses a cross-shaped structure with two 49-element ULAs at the x-axis and y-axis in the range of [−24,24]. The top left and lower right parts are the cross-difference results, which are in fact copies of the original vertical subarray. The starting points of the top left copies are (−7,7), (−8,7), (−9,7), (−10,7), (−11,7), (−16,7), (−21,7), (−26,7) and (−31,7). The FODC configuration is shown in Figure 2b. It is obvious that a URA with the size of 49×49 is located in the center. The URA part can be extracted to perform the 2D spatial smoothing algorithm on it. In this case, at most 49×49 far field uncorrelated sources can be detected, which is much more than that of the existing PSAs based on the SODC concept.

**Remark** **1.** *According to the above analysis, the new structures possess two advantages. First, any 1D subarray geometry having consecutive segments in its self-difference co-array can be adopted. This means that the types of the subarrays can be different, as long as each can generate an equivalent ULA in the self-difference result. This enables us to choose any existing nested-class arrays as needed for further DOF enhancement or mutual coupling reduction. Second, the relative position of the two subarrays can be flexibly set. This is because the cross-shaped section in SODC is generated from the self-difference of subarrays. No matter where the subarrays are located, the difference result would have no change. This inspires us to separate the two subarrays with the parameters $\left(L_{x},L_{y}\right)$ as large as possible, if lower mutual coupling is desired in the GLNA design. When the relative locations grow large enough, the influence between two subarrays can be ignored. Note that in this case, qualitative analysis instead of quantitative analysis for mutual coupling effect is considered. Therefore, we assume that the mutual coupling between subarray can be ignored because of the long relative distance and the mutual coupling inside each subarray is fully calibrated. In the following subsection, we would only compare the DOF of different GLNAs with different types of subarrays.*

### 3.2. DOF Comparisons

For convenience, in this subsection, we set the two subarrays in GLNA as the same type, e.g., the classic nested array [3] and augmented nested array [8]. The minimum DOF that GLNA can obtain is calculated. As a contrast, the DOF of two PSAs, i.e., the hourglass array [16] and 2D nested array [13,14], are compared, as well. Definitions of these structures can be seen in the relative references.

First, assume that subarrays in GLNA-CNA are both classic nested arrays. The number of physical sensors in the dense part and sparse part of the horizontal CNA is $N_{xd}$ and $N_{xs}$. The corresponding values for the vertical CNA are $N_{yd}$ and $N_{ys}$. Take the horizontal CNA as an example. It is the union of the matrices $C_{inner}=\left[1;2;...;N_{xd}\right]$ and $C_{outer}=\left[\left(N_{xd}+1\right);2\left(N_{xd}+1\right);\cdots ;N_{xs}\left(N_{xd}+1\right)\right]$. The CNA can be denoted as:

(11) $$C_{CNA}=\left[C_{inner};C_{outer}\right]=\left[1;2;...;N_{xd};\left(N_{xd}+1\right);2\left(N_{xd}+1\right);\cdots ;N_{xs}\left(N_{xd}+1\right)\right].$$

Its SODC is a ULA with $2N_{xd}\left(N_{xs}+1\right)-1$ elements positioned at $\left[-L_{h},L_{h}\right]=[1-N_{xd}\left(N_{xs}+1\right),$$N_{xd}\left(N_{xs}+1\right)-1]$. Therefore, we have $L_{h}=N_{xd}\left(N_{xs}+1\right)-1$. Similarly, for the vertical CNA, the parameter $L_{v}=N_{yd}\left(N_{ys}+1\right)-1$. According to Property 1, the central URA has the minimum aperture of $\left(2L_{h}+1\right)\times \left(2L_{v}+1\right)$. Therefore, the minimum DOF of GLNA-CNA is:

(12) $${\mathrm{DOF}}_{\mathrm{GLNA}-\mathrm{CNA}}=\left[2N_{xd}\left(N_{xs}+1\right)-1\right]\left[2N_{yd}\left(N_{ys}+1\right)-1\right].$$

For the GLNA with augmented nested arrays as subarrays (GLNA-ANA), we use ANAI-2 [8] as the subarrays. For ANAI-2, its definition is:

(13) $$C_{ANAI-2}=\left[L_{1};M_{1};R_{1}\right]$$

with $L_{1}=\left[1;2;4;\cdots ;2k_{1}+2\right]$, $M_{1}=\left[0;\left(N_{d}+1\right);2\left(N_{d}+1\right);\cdots ,\left(N_{s}+1\right)\left(N_{d}+1\right)\right]$, $R_{1}=[\left(N_{d}+1\right)$$N_{s}+N_{d}-2;\left(N_{d}+1\right)N_{s}+N_{d}-4;\cdots ;\left(N_{d}+1\right)N_{2}+N_{d}-2k_{2};\cdots ;\left(N_{d}+1\right)N_{s}]$. Here, $N_{d}$ and $N_{s}$ are the sensor numbers of the dense part and sparse part of its original classic nested array, respectively. $k_{1}$ and $k_{2}$ are the parameters with $k_{1}\in \left[0,R\left\lfloor \left(N_{1}-1\right)/2\right\rfloor \right]$ and $k_{2}\in \left[1,R\left\lfloor N_{1}/2\right\rfloor \right]$. R⌊α⌋ round the value to the nearest integer, where R⌊α⌋≤α. In this case, ANAI-2 instead of other kinds of augmented nested array is chosen, as it has similar parameters as other nested arrays, so that it is easier for us to compare.

Assume the array parameters as $\left(N_{xd},N_{xs}\right)$, which denote $\left(N_{d},N_{s}\right)$ on the x-axis, and $\left(N_{yd},N_{ys}\right)$m which is $\left(N_{d},N_{s}\right)$ in the y-axis direction. Based on the properties in [8], it is not difficult to find that the consecutive parameters in SODC are $L_{h}=\left(N_{xd}+1\right)N_{xs}+N_{xd}-3$ and $L_{v}=\left(N_{yd}+1\right)N_{ys}+N_{yd}-3$. Therefore, the minimum DOF of GLNA-ANA is:

(14) $${\mathrm{DOF}}_{\mathrm{GLNA}-\mathrm{ANA}}=\left[2\left(N_{xd}+1\right)N_{xs}+2N_{xd}-5\right]\left[2\left(N_{yd}+1\right)N_{ys}+2N_{yd}-5\right].$$

For fair comparisons, we consider the 2D nested array (2D-NA) and perform the fourth-order difference algorithm on it for DOF analysis. Denote $N_{xd}$ and $N_{xs}$ as the number of dense and sparse elements on the x-axis. $N_{dy}$ and $N_{sy}$ are the corresponding parameters on the y-axis. Then, the dense and sparse URAs in 2D-NA have $N_{xd}N_{yd}$ and $N_{xs}N_{ys}$ physical sensors, respectively. According to the results in [13,14], the SODC of 2D-NA is a URA with the size of $N_{xd}N_{xs}\times \left(2N_{yd}N_{ys}-1\right)$. Perform the difference again. Then, the URA region in FODC has the aperture of:

(15) $${\mathrm{DOF}}_{2D-\mathrm{NA}}=\left(2N_{xd}N_{xs}-1\right)\times \left(4N_{yd}N_{ys}-3\right).$$

For the hourglass array (HA), the related definition and properties are provided in [16]. Consider $N_{x}$ sensors located in the horizontal direction. $2N_{y}-2$ sensors are distributed on the vertical lines. Then, its SODC is proven to be a URA with the size of $\left(2N_{x}-1\right)\left(2N_{y}-1\right).$ The FODC is definitely a URA with the DOF of:

(16) $${\mathrm{DOF}}_{\mathrm{HA}}=\left(4N_{x}-3\right)\left(4N_{y}-3\right).$$

For better understanding, we take the aforementioned PSAs with the same number of sensors which is 18 as an example. The minimum DOFs of GLNAs and other two PSAs are summarized in Table 1. The relative position of two subarrays in GLNAs is set as $\left(L_{x},L_{y}\right)=\left(-6,6\right)$. As can be seen, all GLNAs can acquire a much higher number of DOF than the other two PSAs. This is because 2D-NA and HA are proposed for second-order difference design. When the fourth-order difference concept is applied on the two PSAs, much difference redundancy exists. As for the two kinds of GLNAs, the GLNA-ANA has the largest URA in FODC, since ANAI-2 has more consecutive lags in SODC than CNA. Meanwhile, GLNA-CNA is a little worse than the GLNA-ANA. The better the 1D sparse array we choose, the higher the DOF we obtain.

## 4. A Special GLNA Configuration with Enhanced DOF

For convenient analysis, GLNAs discussed in the above subsection are chosen as PSAs with the same type of subarrays. However, for the generalized structure defined by (9), the two subarrays are allowed to be totally different types. In this subsection, we will provide a special GLNA with a nested array and an augmented nested array, ANAI-2, as subarrays. By setting their relative position $\left(L_{x},L_{y}\right)$ as specific values, the special GLNA can acquire a much higher DOF than the aforementioned GLNAs. We use sGLNA to represent this kind of GLNA in this paper.

First, observe the SODC structure of GLNA-CNA in Figure 2a. The virtual ULAs $D_{h}$ and $D_{v}$ on the x-axis and y-axis are generated from the self-difference of subarrays. As long as the nested-class arrays are chosen, the cross-shaped ULAs can be obtained. For the sparse elements in the second and fourth quadrants, they come from the cross-difference $D_{vh}$ and $D_{hv}$. The location of this part is determined by the relative position $\left(L_{x},L_{y}\right)$. Note that in the definition of GLNA in the above section, $L_{x}$ and $L_{y}$ are set to be arbitrary integers. However, if $\left(L_{x},L_{y}\right)$ is chosen as special values, the cross-difference parts will probably move to make one of the consecutive lags in this area connected to the virtual ULA at the y-axis. As a result, the length of the cross-shaped structure in the y-direction will be extended. The size of FODC will be enlarged, as well.

Motivated by the above strategy, we use one of the four augmented nested array structures called ANAI-2 [8] as the subarray in the x-direction and another classic nested array in the y-direction to form sGLNA. Here, ANAI-2 instead of other kinds of augmented nested arrays is chosen as it has similar parameters as other nested arrays, so that it is easier for us to compare. In order to define this special GLNA, $\left(L_{x},L_{y}\right)$ are chosen as special values. $L_{y}=N_{ys}\left(N_{yd}+1\right)-1$, and let the value of $L_{x}$ be equal to the abscissa of subarray H, i.e., $L_{x}$ is an element of $S_{h}$ Then, we have the following property.

**Property** **2.** *For sGLNA with ANAI-2 and classic nested array being the horizontal and vertical subarray having parameters $N_{xd},N_{xs}$ and $N_{yd},N_{ys}$, the cross-shaped structure in SODC is located at $\left[S_{v};S_{h}\right]$ with $S_{v}=\left[\left(0,-L_{v}\right);\left(0,-L_{v}+1\right);\cdots ;\left(0,L_{v}-1\right);\left(0,L_{v}\right)\right]$ where $L_{v}=N_{ys}\left(N_{yd}+1\right)+N_{yd}-1$ and $S_{h}=\left[\left(-L_{h},0\right);\left(-L_{h}+1,0\right);\cdots ;\left(L_{h}-1,0\right);\left(L_{h},0\right)\right]$ where $L_{h}=\left(N_{xd}+1\right)N_{xs}+N_{xd}-3$. The size of FODC is $\left[2\left(N_{xd}+1\right)N_{xs}+2N_{xd}-5\right]\times \left[2N_{ys}\left(N_{yd}+1\right)+2N_{yd}-1\right]$.*

**Proof.**  Since the horizontal subarray is an ANAI-2, the 1D physical sensors’ locations are given according to the definition of augmented nested arrays in [8] and Equation (13). According to [8], its self-difference is a hole-free ULA. Therefore, $D_{h}=S_{h}=\left[\left(-L_{h},0\right);\left(-L_{h}+1,0\right);\cdots ;\left(L_{h}-1,0\right);\left(L_{h},0\right)\right]$ with $L_{h}=\left(N_{xd}+1\right)N_{xs}+N_{xd}-3$.

For the vertical subarray, as it is a nested array, the 1D sensor location is $C_{v}=\left[L_{1};R_{1}\right]$ with $L_{1}=\left[1;2;\cdots ;N_{yd}\right]$ and $R_{1}=\left[\left(N_{yd}+1\right);2\left(N_{yd}+1\right);\cdots ;N_{ys}\left(N_{yd}+1\right)\right]$. Its self-difference is hole-free, as well, with the one-side consecutive length as $L_{v1}=N_{ys}\left(N_{yd}+1\right)-1$.

Now, let us see the second-order cross-difference $D_{vh}$. From the analysis in the proof of Property 1, we have the $D_{vh}$ is a matrix, the elements of which can be denoted as $\left(-s_{h,i}+L_{x},s_{v,j}+L_{y}\right)$. Here, $s_{h,i}$ and $s_{v,j}$ are the *i*-th and the *j*-th elements of the matrices $C_{h}$ and $C_{v}$, respectively. Since for sGLNA, $L_{x}$ is an element of $C_{h}$ and $L_{y}=N_{ys}\left(N_{yd}+1\right)-1$, it can be deduced that $L_{x}$ must be equal to one of the horizontal coordinates of ANAI-2. Therefore, there must be cross difference array in SODC, the *j*-th element of the matrix of which can be denoted as $\left(0,s_{v,j}+L_{y}\right)$, which is located on the y-axis. As a ULA part exists in the $C_{v}$, the consecutive lags in the above matrix will become $\left[\left(0,N_{ys}\left(N_{yd}+1\right)\right);\left(0,N_{ys}\left(N_{yd}+1\right)+1\right);\left(0,N_{ys}\left(N_{yd}+1\right)+2\right);\cdots ;\left(0,N_{ys}\left(N_{yd}+1\right)+N_{yd}-1\right)\right]$. As a result, the consecutive length is $L_{v2}=N_{yd}$. Combined with the self-difference result, the consecutive length in the vertical direction is $L_{v}=L_{v1}+L_{v2}=N_{ys}\left(N_{yd}+1\right)-1+N_{yd}$.

As for FODC, URA is in the part $\left(2L_{v}+1\right)\times \left(2L_{h}+1\right)$. Therefore, the size of URA is $\left[2\left(N_{xd}+1\right)N_{xs}+2N_{xd}-5\right]\times \left[2N_{ys}\left(N_{yd}+1\right)+2N_{yd}-1\right]$. ☐

**Figure 3:**
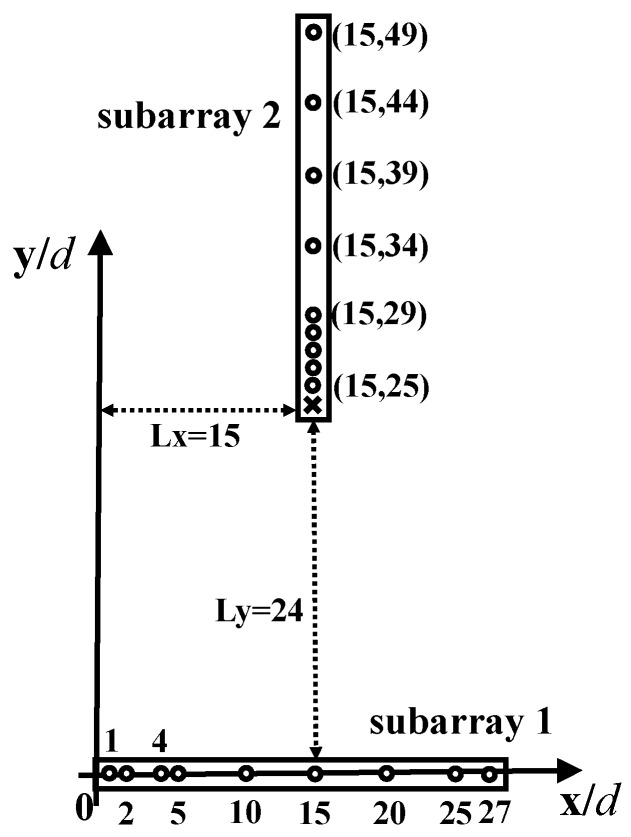
An example of sGLNA with $\left(N_{xd},N_{xs},N_{yd},N_{ys}\right)=\left(4,5,4,5\right)$ and $\left(L_{x},L_{y}\right)=\left(15,24\right)$. The circles represent physical sensors. The cross is a hole.

Figure 3 illustrates an sGLNA with the horizontal subarray as an ANAI-2 having parameters as $\left(N_{xd},N_{xs}\right)=\left(4,5\right)$. The vertical subarray is a nested array with parameters as $\left(N_{yd},N_{ds}\right)=\left(4,5\right)$. The relative position is $\left(L_{x},L_{y}\right)=\left(15,24\right)$. As can be seen, the subarray 2 is aligned with one of the sensors in Subarray 1. Such an arrangement guarantees that one copy of Subarray 2 is located at the y-axis in SODC, which can connect to the virtual ULA from 1 to 24, generated from the self-difference of Subarray 2. As such, the consecutive lags extend from the original range of [−24,24] to [−29,29]. Since Subarray 1 is an ANAI-2, its self-difference is hole-free, located in the range of [−26,26]. We obtain that the URA in FODC has dimensions of (2×26+1)(2×29+1)=3127.

For comparison, the DOF of sGLNA is listed in Table 1. We conclude that sGLNA has the highest number of DOFs among all GLNAs. That is because one of subarrays in sGLNA is ANAI-2, which possesses the best performance in DOF enhancement. Besides, another subarray is a nested array. Although its DOF is less than that of ANAI-2, it has a dense ULA part, which can be used to enlarge the consecutive range in the cross-shaped structure of SODC. The values of $L_{x}$ and $L_{y}$ make sure that the copy of the nested array can be connected to the virtual ULA at the y-axis in SODC. The extended consecutive range leads to the great enhancement of DOF.

## 5. Numerical Experiments

In this section, simulation results are presented to compare the performance of GLNA-CNA, GLNA-ANA and sGLNA. The hourglass array and 2D nested array are also simulated as a contrast. In the experiments, we assume the number of sensors to be 18. The parameters for GLNA-CNA, GLNA-SNA, GLNA-ANA and sGLNA are $\left(N_{xd},N_{xs},N_{yd},N_{ys}\right)=\left(4,5,4,5\right)$. The relative position is defined by $L_{x}$ and $L_{y}$. $\left(L_{x},L_{y}\right)=\left(5,29\right)$. For the 2D nested array and the hourglass array, the configurations are quite different from GLNAs. To make sure the total number of sensors is the same as that of GLNAs, we choose the parameters as $\left(N_{dx},N_{dy},N_{sx},N_{sy}\right)=\left(3,3,3,3\right)$ and $\left(N_{x},N_{y}\right)=\left(6,7\right)$, respectively. The QS signals with P=10 frames are used.

**Figure 4:**
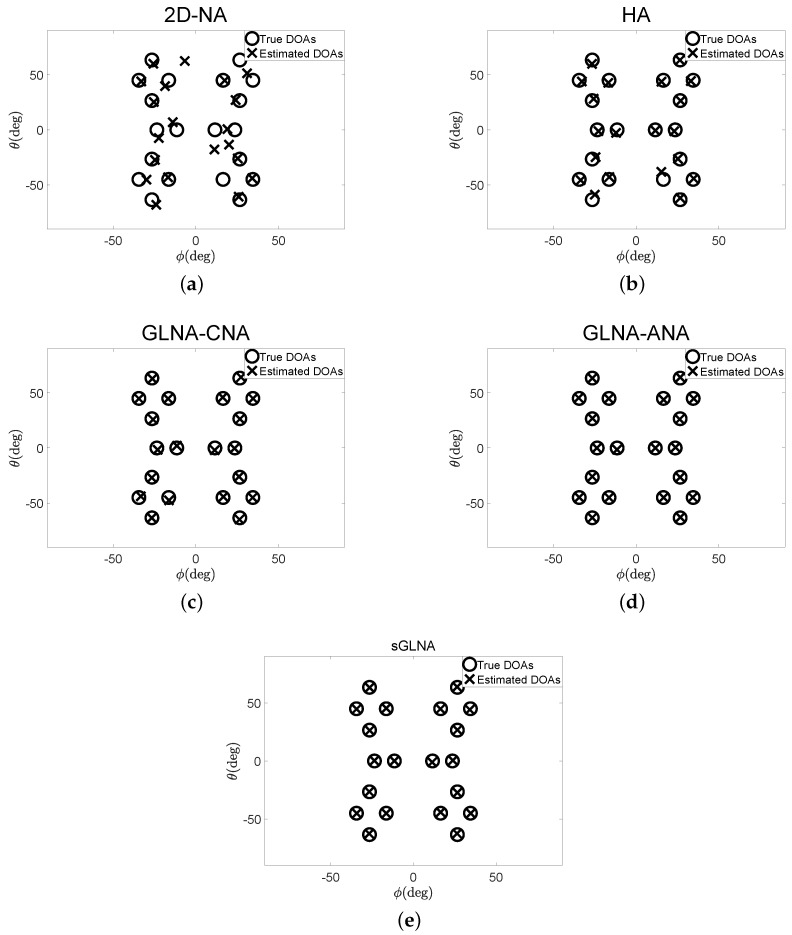
DOA estimations of different PSAs (**a**) 2D-NA, RMSE = 8.0128${}^{\circ }$; (**b**) HA, RMSE = 2.5737${}^{\circ }$; (**c**) GLNA-CNA, RMSE = 1.1827${}^{\circ }$; (**d**) GLNA-ANA, RMSE = 0.5488${}^{\circ }$; (**e**) sGLNA, RMSE = 0.5441${}^{\circ }$.

In the first experiment, D=20 uncorrelated sources with the directions (θ,ϕ) are distributed at (−34${}^{\circ }$,45${}^{\circ }$), (−26${}^{\circ }$,63${}^{\circ }$), (26${}^{\circ }$,−63${}^{\circ }$), (34${}^{\circ }$,−45${}^{\circ }$), (−26${}^{\circ }$,26${}^{\circ }$), (−16${}^{\circ }$,45${}^{\circ }$), (16${}^{\circ }$,−45${}^{\circ }$), (26${}^{\circ }$,−26${}^{\circ }$), (0${}^{\circ }$,−23${}^{\circ }$), (0${}^{\circ }$,−11${}^{\circ }$), (0${}^{\circ }$,11${}^{\circ }$), (0${}^{\circ }$,23${}^{\circ }$), (−26${}^{\circ }$,−26${}^{\circ }$), (−16${}^{\circ }$,−45${}^{\circ }$), (16${}^{\circ }$,45${}^{\circ }$), (26${}^{\circ }$,26${}^{\circ }$), (−34${}^{\circ }$,−45${}^{\circ }$), (−26${}^{\circ }$,−63${}^{\circ }$), (26${}^{\circ }$,63${}^{\circ }$), (34${}^{\circ }$,45${}^{\circ }$). They are assumed to impinge on 2D-NA, HA, GLNA-CNA, GLNA-ANA and sGLNA. These source angles are selected randomly. The SNR is 20 dB. $\tilde{P}=100$ snapshots in each frame, in total $K=\tilde{P}\times P=1000$, are sampled for $R_{xx}\left(p\right)$ construction. The DOA estimation results are examined and compared in Figure 4. For each configuration, the root mean square error (RMSE) is defined as:

(17) $$\mathrm{RMSE}=[\left(1/\left(D\tilde{N}\right)\sum_{n=1}^{\tilde{N}}\sum_{i=1}^{D}{\left(\hat{\theta_{in}}-\theta_{in}\right)}^{2}+{\left(\hat{\phi_{in}}-\phi_{in}\right)}^{2}\right]{}^{1/2}$$

where $\left(\hat{\theta },\hat{\phi }\right)$ and (θ,ϕ) are the estimated DOAs and the true DOAs; $\tilde{N}$ is the number of trials. In this experiment, $\tilde{N}=30$. As can be concluded from Figure 4a, 2D-NA fails to estimate some directions with RMSE = 8.0128${}^{\circ }$. The situation becomes better for HA. However, there are still obvious estimation errors with RMSE = 2.5737${}^{\circ }$. In contrast, as Figure 4c–e illustrate, all GLNAs can estimate DOAs correctly. The sGLNA has the lowest RMSE = 0.5441${}^{\circ }$, compared to the GLNA-CNA with RMSE = 1.1827${}^{\circ }$ and GLNA-ANA with RMSE = 0.5488${}^{\circ }$.

**Figure 5:**
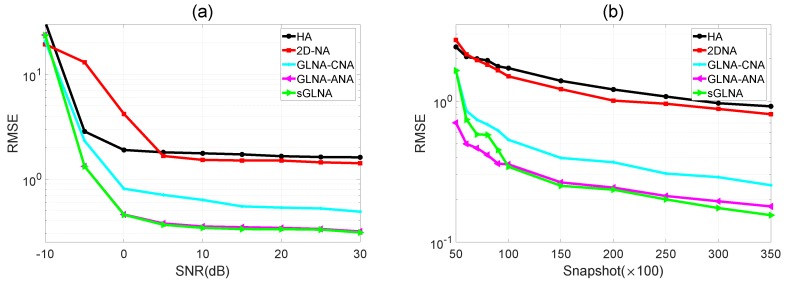
DOA estimation comparisons. (**a**) RMSE as a function of SNR; (**b**) RMSE as a function of snapshot.

In the second experiment, we compare the RMSE results of the five kinds of PSAs mentioned above at different SNRs and snapshots. D=20 sources distributed in the same normalized directions as the first experiment are used in this experiment. Figure 5a depicts the RMSE versus SNR results. $\tilde{N}=100$ independent trials are conducted for each SNR. The snapshot number is set as K=100×100. The SNR varies from −10 dB to 30 dB. Figure 5b provides the RMSE comparisons with the snapshot number varying from 50×100 to 350×100. The SNR is 10 dB in this case. As can be seen, for both figures, that sGLNA has the lowest estimation error among all five sparse arrays. This is because sGLNA can acquire the highest number of DOF. For other PSAs, GLNA-ANA has a slightly worse performance than sGLNA, followed by GLNA-CNA, 2D-NA and HA. Therefore, GLNAs can achieve better DOA performance than the second-order-based PSAs. This result coincides with the conclusion in Table 1. The reason why sGLNA is less robust than ALNA-ANA in Figure 5b is that the robustness performance of one PSA is determined by the distribution of its difference redundancy. Note that for sGLNA, it has 12 redundancy functions not less than two, and for GLNA-ANA, it has 14 redundancy functions not less than two. ALNA-ANA has more redundancy. Therefore, it is more robust than sGLNA.

## 6. Conclusions

In this paper, a generalized L-shaped nested array framework is proposed. The expressions of the dimensions of the cross-shaped structure in SODC and URA size in FODC are provided and verified.

Several GLNAs, such as GLNA-CNA and GLNA-ANA, are analyzed and compared in the DOF aspects. For DOF improvement, a special GLNA configuration is proposed and compared to other PSAs. In conclusion, GLNAs enjoy closed-form sensor locations and large URA sections in FODC. They can acquire much higher DOFs than any other SODC-based PSAs by choosing the subarrays and setting the distance between them properly. Meanwhile, note that for the GLNA framework, as a sparse array structure can be constructed and a low difference redundancy can be achieved, the mutual coupling effect is reduced and can be quantitatively analyzed in the future. Several numerical experiments indicate that these structures perform better than the existing PSAs.

## Figures and Tables

**Table 1:** Comparisons of DOF with the number of sensors as N=18. HA, hourglass array; ANA, augmented nested array; sGLNA, special GLNA.

Array Structures	Parameters	DOF
HA	$\left(N_{x},N_{y}\right)=\left(6,7\right)$	525
2D-NA	$\left(N_{xd},N_{xs},N_{yd},N_{ys}\right)=\left(3,3,3,3\right)$	561
GLNA-CNA	$\left(N_{xd},N_{xs},N_{yd},N_{ys}\right)=\left(4,5,4,5\right)$	2401
GLNA-ANA	$\left(N_{xd},N_{xs},N_{yd},N_{ys}\right)=\left(4,5,4,5\right)$	2809
sGLNA	$\left(N_{xd},N_{xs},N_{yd},N_{ys}\right)=\left(4,5,4,5\right)$	3127
